# Sexual-related determinants of life satisfaction among married women: A cross-sectional study

**DOI:** 10.1186/s12905-023-02365-5

**Published:** 2023-04-28

**Authors:** Nasim Bahrami, Mobina Hosseini, Mark D. Griffiths, Zainab Alimoradi

**Affiliations:** 1 Social Determinants of Health Research Center, Research Institute for Prevention of Non- Communicable Diseases, Qazvin University of Medical Sciences, Qazvin, 34197-59811 Iran; 2 Students’ Research Committee, Qazvin University of Medical Sciences, Qazvin, Iran; 3 International Gaming Research Unit, Psychology Department, Nottingham Trent University, Nottingham, UK

**Keywords:** Life satisfaction, Married women, Intimacy, Sexual functioning, Sexual distress, Dyadic sexual communication

## Abstract

**Background and aim:**

Life satisfaction refers to the perceived satisfaction of individuals concerning various aspects of their lives. The present study investigated the predictive role of sexual-related determinants in life satisfaction among married women.

**Methods:**

A cross-sectional study was conducted from August to November 2021. A total of 350 married women with at least six months of cohabitation with husbands were included in the study. The study utilized a multi-stage random sampling method from 10 comprehensive health centers in Qazvin, Iran. Scores on the Emotional Intimacy Questionnaire (EIQ), Dyadic Sexual Communication Scale (DSCS), Female Sexual Distress Questionnaire (FSDQ), Female Sexual Quality of Life Scale (FSQLS), Female Sexual Function Index (FSFI), and Life Satisfaction Scale (LSS) were assessed. Data were analyzed using univariable and multivariable linear regression models with a significance level of *p* < 0.05.

**Results:**

The mean age of participants was 33.77 years (SD = 9.77) and they had been married for an average of 10.21 years (SD = 9.93). The mean scores on the LSS were 20.16 (out of 35; SD = 6.79). Based on the multivariable linear regression model adjusted for socio-demographic characteristics, the two strongest predictors of life satisfaction among Iranian married women were marital intimacy (β = 0.49, *p* < 0.001) and sexual functioning (β = 0.17, *p* = 0.009). Together, these variables explained 45% of variance in life satisfaction.

**Conclusion:**

Given that marital intimacy and sexual functioning were the most significant sexual-related determinants of life satisfaction among married women, designing and implementing interventions which increase women’s marital intimacy and sexual functioning might improve married women’s life satisfaction.

## Introduction

‘Life satisfaction’ as a general term that describes the perceived satisfaction of individuals concerning various aspects of their lives, and is the extent to which individuals’ basic needs have been met [1]. In fact, life satisfaction is a positive assessment of individuals’ whole lives [2]. Factors affecting life satisfaction can be classified into two main categories comprising biological factors (such as genetic factors and physical health) and socio-psychological factors (such as desirable social relationships and psychological needs) [3].

One of the psychosocial factors that can affect individuals’ life satisfaction is marriage [4]. In many cultures (such as Iran, where the present study was carried out), the relationship between a man and a woman is the longest relationship between the two opposite sexes and the continuation of marital relations is at the core of the family system. Consequently, personal satisfaction with the marital relationship is considered very important [5]. Sexual-related aspects are an important aspect of marital relationship with different and inter-related dimensions such as sexual functioning [6, 7], sexual communication [8], sexual problems [9], sexual quality of life [10], sexual satisfaction [11], and intimacy [12].

Satisfactory sexual functioning is characterized by the lack of problems in the stages of sexual desire, arousal and orgasm, as well as subjective satisfaction with the frequency and outcome of individual and partner sexual behavior [13]. Sexual functioning is a complex and dynamic interaction of physical, emotional and psychosocial states [14]. The understanding of sexual functioning is influenced by the current state of medical knowledge and the social values maintained within respective cultures [15]. Emotional, verbal, and physical closeness are formed through intimate relationships between couples, and brings satisfaction to their marital relationship [16]. Couples’ dyadic sexual communication (i.e., sexual conversation) can independently predict marital and sexual satisfaction [8, 17–19].

Emotional intimacy between couples can also be effective in improving life satisfaction [20]. The quality of the couple’s intimate relationship is an important part of their relationship and sexual satisfaction [21]. Emotional intimacy refers to the general quality of a person’s closeness with their life partner whereas sexual intimacy refers to the degree of closeness of a person with their life partner during sex, both of which are important in the level of sexual relationship satisfaction [22].

Sexual distress is one of the factors that can have a negative impact on the quality of sexual functioning and, consequently, on the quality of married life among couples [23]. There is a relationship between increased sexual distress and decreased satisfaction with sexual relationships [24]. Research has indicated that women generally suffer from more sexual distress than men [25]. In general, sexual distress is related to the quality of a person’s sexual life which, in turn, is related the person’s satisfaction with their sexual life [26].

Sexual quality of life is viewed as one of the indicators of sexual health [27]. Sexual health is one of the basic and important factors in the stability of married life. One of the most important factors of couples’ happiness and good quality of life is good sexual relations. It also has a positive effect on a person and improves self-confidence and relationships [28]. Sexual life is one of the key issues in the field of sexual and reproductive health and is also an important aspect of women’s quality of life [29]. Research conducted in this field has examined the impact of factors such as employment [30], socio-economic issues [31], religious attitudes [32], socio-cultural issues [33] on life satisfaction and marital satisfaction. In other studies, the relationship between different components of sexual life with each other has been investigated [10, 34].

However, to best of the present authors’ knowledge, the relationship between sexual-related determinants of life satisfaction has not previously been investigated, and prior studies have been more focused on the impact of sexual-related determinants in relation to the marital satisfaction of couples. Although there are many different variables that can be investigated regarding the sexual life of individuals, the present study examined some of the most important variables. The effect of these variables together in the context of Iranian culture has not been properly examined in previous studies. Therefore, the present study examined these variables together in a regression model to identify the variables which most affected women’s life satisfaction. Considering the importance of culture in examining various issues affecting individual’s life satisfaction, the present study examined the predictive role of important sexual-related determinants including sexual functioning, emotional intimacy, dyadic sexual communication, sexual distress, and sexual quality of life, in relation to life satisfaction among Iranian married women.

## Methods

### Participants, design, and procedure

The present cross-sectional study was conducted from August to November 2021. Married women with at least six months of cohabitation with husbands were included in the study. The exclusion criteria included unwillingness to participate in the study, having (self-reported) physical and psychological illnesses, experiencing stressful events in the past three months (including loss of loved ones), being pregnant, currently lactating (less than six months), being divorced or being widowed. The sample size for linear multivariable analysis was estimated 350 people based on Sawyer and Ball’s general rule [35] (n = 12 + 11k, k = 30 predictors). Recruitment comprised a multi-stage random sampling method. At first, Qazvin city was divided into five clusters based on geographical regions, and two comprehensive health centers were selected randomly from each cluster. Then, 35 married women from each center were selected randomly. After receiving the telephone numbers of potential participants from their health records, they were contacted. After stating the objective of the study and the methods, if individuals were willing to participate in the study, an online link to participate in the survey that was hosted on the *PorsLine* platform was sent to them via *WhatsApp* or SMS. On the first page of the survey, they were asked to provide their consent to participate in the study. If they consented, they were able to complete the survey. Informed consent was required orally to send the survey link and written informed consent was provided in first page of survey link. To provide their consent to participate, individuals were asked to click on an ‘agree’ button to continue the survey.

### Measures

#### Demographic and Reproductive Characteristics Checklist

This checklist contained questions concerning the woman’s age, education and occupation, her spouse’s age, education and occupation, marriage duration, perceived family economic status, having an independent bedroom, having independent living conditions, monthly number of sexual coitus, the number of pregnancies, number of living children, and contraceptive methods used.

#### Life Satisfaction Scale (SWLS)

The five-item Persian version of SWLS [36] was used. The scale assesses the cognitive component of actual well-being (e.g., how satisfied individuals are with their lives or how close individuals are to their ideal life). Each item is rated on a seven-point response scale from 1 (*completely disagree*) to 7 (*completely agree*). The total score is obtained from the sum of the scores of each item and scores range from 5 to 35. A higher score indicates greater life satisfaction [36]. In the present study, the internal consistency of the scale was 0.90 based on Cronbach’s alpha.

#### Female Sexual Quality of Life Questionnaire (FSQLS)

The 18-item Persian version of the FSQLS [37] was used. The items (e.g., *“When I think about my sex life, I feel depressed”*) are rated on a six-point response scale from 1 (*completely agree*) to 6 (*completely disagree*). The total score is obtained from the sum of the scores of each item and scores range from 18 to 108. Higher scores indicate a better quality of sex life for women. In the present study, the internal consistency of the scale was 0.95 based on Cronbach’s alpha.

#### Female Sexual Distress Questionnaire (FSDQ)

The 13-item Persian version of Revised FSDS was used [38]. The items that examine different aspects of female distress related to sexual activity (e.g., *“In the past 4 weeks, how often have you been anxious about your sex life?”*) are rated on a five-point response scale from 0 (*never*) to 4 (*always*). The total score is obtained from the sum of the scores of each item and scores range from 0 to 52. A higher score indicates greater sexual distress. In the present study, the internal consistency of the scale was 0.94 based on Cronbach’s alpha.

#### Emotional Intimacy Questionnaire (EIQ)

The 17-item Persian version of EIQ was used [39]. Items (e.g., *“We want to spend time together”*) are rated on a seven-point response scale from 1 (*never*) to 6 (*almost always*). The total score is obtained from the sum of the scores of each item and scores range from 17 to 119. Higher scores indicate greater intimacy. In the present study, the internal consistency of the scale was 0.98 based on Cronbach’s alpha.

#### Dyadic Sexual Communication Scale (DSCS)

The 13-item Persian version of the DSCS [8] was used. Items (e.g., *“There are issues or problems in our sexual relationship that we have never talked about them”*) are rated on a six-point response scale from 1 (*strongly disagree*) to 6 (*strongly agree*). The total score is obtained from the sum of the scores of each item and scores range from 13 to 78. Higher scores indicate better dyadic sexual communication [8]. In the present study, the internal consistency of the scale was 0.91 based on Cronbach’s alpha.

*Female Sexual Function Index (FSFI)*: The six-item Persian version of the FSFI was used [40]. The six-item version is a shorter form of the original 19-item FSFI [41]. The scale assesses various aspects of women’s sexual function: desire, arousal, vaginal moisture, orgasm, sexual satisfaction, and intercourse pain [41]. Items (e.g., *“When you had sexual stimulation or intercourse, how often did you reach orgasm?”*) are rated on a five-point response scale from 1 (*Almost never or never*) to 5 (*Almost always or always*). The total score is obtained from the sum of the scores of each item and scores range from 2 to 30. Higher scores indicate better sexual functioning. Moreover, the combination of these six items have been shown to be the best and most valid scale to identify women with sexual dysfunction. In the present study, the internal consistency of the scale was 0.84 based on Cronbach’s alpha.

### Ethics

The present study was approved by the Institutional review board and Regional Ethics Committee of Biomedical Research (decree code: IR.QUMS.REC.1400.239). All methods were carried out in accordance with relevant guidelines and regulations. When conducting the research, the necessary permits were obtained to attend comprehensive health centers. The design, objectives, and research methods were fully explained to the participants, and informed consent was obtained from individuals to participate in the study. Individuals were assured that their information would remain confidential. Voluntary participation in the study and having the full authority of the participants to continue or withdraw from the study was observed.

### Data analysis

Data were analyzed using SPSS software version 24. Categorical variables are described with frequencies and percentages and continuous variables are described with means and standard deviations. The association of socio-demographic characteristics and sexual-related determinants (including emotional intimacy, dyadic sexual communication, female sexual distress, female sexual quality of life, and female sexual function) with life satisfaction was assessed using univariable linear regression analysis. Based on the results of univariable regression, significant variables were entered in multivariable regression model. Multivariable linear regression model was then used to examine the predictive role of sexual-related determinants for life satisfaction. Life satisfaction score was entered as the dependent variable and the sexual-related determinants were entered as the independent variables utilizing the stepwise approach. Assumptions of linear regression model (including checking the normality of life satisfaction and the absence of controlled outliers) were tested. After performing regression analyses, VIF < 2 and tolerance < 1 confirmed the possibility for using the regression model. The Durbin-Watson Index for the whole model was 1.65. Therefore, the assumptions of the linear regression model were established. The significance level of all tests was *p* ≤ 0.05.

## Results

In the present study, 350 women with a mean age of 33.77 years participated (SD = 9.77). The mean scores were 20.16 (out of 35) for LSS (SD = 6.79), 79.82 (out of 108) for the FSQLS (SD = 19.11), 14.35 (out of 52) for the FSDQ (SD = 10.89), 87.29 (out of 119) for the EIQ (SD = 24.27), 21.33 (out of 30) for the FSFI (SD = 5.06), and 53.38 (out of 78) for the DSCS (SD = 13.44). (Table 1). Based on multivariable linear regression model (Table 2), marital intimacy was the strongest predictor of life satisfaction (β = 0.42, *p* < 0.001). Moreover, sexual function (β = 0.14, *p* = 0.009) and dyadic sexual communication (β = 0.14, *p* = 0.05) were other significant predictors of life satisfaction among Iranian married women. These variables together explained 43% of the variance in life satisfaction. After controlling for socio-demographic characteristics, the two strongest predictors of life satisfaction among married Iranian women were marital intimacy (β = 0.49, *p* < 0.001) and sexual functioning (β = 0.17, *p* = 0.009). Together, these variables explained 45% of variance in life satisfaction.

**Table 1:** Distribution of socio-demographic variables

	Range	Mean (SD)
Age (in years)	18–63	33.77 (9.77)
Spouse’s age (in years)	22–69	37.67 (9.72)
Spousal age difference (in years)	Spouse age minus women’s age	3.90 (3.76)
Marriage duration (in years)	1–43	10.21 (9.93)
Number of children	0–6	1.12 (1.16)
Coitus monthly	0–48	7.93 (6.24)
Female Sexual Quality of Life Scale total	23–108	79.82 (19.11)
Female Sexual Distress Questionnaire total	0–52	14.35 (10.89)
Emotional Intimacy Questionnaire total	17–119	87.29 (24.27)
Female Sexual Function Index total	5–29	21.33 (5.06)
Dyadic Sexual Communication Scale total	18–78	53.38 (13.44)
Life Satisfaction Scale total	5–35	20.16 (6.79)
Categorical variables		N (%)
Education	Under Diploma	47 (13.4)
	Diploma	118 (33.7)
	Academic	185 (52.9)
Spouse education	Under Diploma	44 (12.6)
	Diploma	89 (25.4)
	Academic	217 (62.0)
Job	Employed	163 (46.6)
	Housewife	187 (53.4)
Spouse’s job	Employed	306 (87.4)
	Retired	29 (8.3)
	Unemployed	15 (4.3)
Economic status	Poor	37 (10.6)
	Fair	214 (61.1)
	Good	99 (28.3)
Having separate bedroom	No	68 (19.4)
	Yes	282 (80.6)
Living condition	With family	48 (13.7)
	Independent	302 (86.3)
Using contraception	No	158 (45.1)
	Yes	192 (54.9)

**Table 2:** Results of multivariable linear regression analysis (via stepwise method) assessing the predictors of life satisfaction

		Unstandardized Coefficients		Standardized Coefficients	Sig.	Collinearity Statistics	
		B (95% CI)	Std. Error	Beta		Tolerance	VIF
Model 1	Intimacy	0.12 (0.08; 0.16)	0.02	0.42	< 0.001	0.38	2.65
	FSFI	0.21 (0.05; 0.37)	0.08	0.16	0.009	0.45	2.23
	DCSS	0.07 (0.00; 0.14)	0.04	0.14	0.05	0.35	2.85
Model 2	Intimacy	0.14 (0.11; 0.17)	0.02	0.49	< 0.001	0.53	1.89
	FSFI	0.22 (0.08; 0.37)	0.07	0.17	0.003	0.52	1.94
Model summary: Model 1: Independent Variable: Coitus Monthly; S-QoL; FSD; Intimacy; FSFI; DSC; Adjusted R Square: 0.43; Durbin-Watson: 1.72 Model 2: Independent Variable: Coitus Monthly; S-QoL; FSD; Intimacy; FSFI; DSC and scio demographic variables; Adjusted R Square: 0.45; Durbin-Watson: 1.65 FFSI = Female Sexual Function Index. DCSS = Dyadic Sexual Communication Scale							

## Discussion

The purpose of the present study was to determine the predictive role of sexual-related determinants in life satisfaction among married Iranian women. Previous studies have investigated the association between life satisfaction and some non-sexual determinants such as relationship commitment [20], stress [30], socio-economic factors [31, 42], social and cultural factors [33], and social health [43]. Studies have occasionally assessed the association between sexual life variables with sexual and marital satisfaction [17, 18] but not life satisfaction. Moreover, sexual self-disclosure has been found to be associated with sexual satisfaction [18], while communication and sexual satisfaction have been associated with marital satisfaction [17]. Most of these studies have assessed uni-variable associations. Therefore, the present study examined these variables together in a regression model to identify the variables most affecting women’s life satisfaction. More specifically, the study examined the predictive role of sexual-related determinants including sexual functioning, emotional intimacy, dyadic sexual communication, sexual distress, and sexual quality of life, in relation to life satisfaction among married Iranian women.

Based on the results of the study’s multivariable regression model examining the role of sexual-related determinants in life satisfaction, two variables were identified as significant predictors of women’s life satisfaction (i.e., good marital intimacy and high sexual functioning). Marital intimacy was the strongest predictor of women’s life satisfaction in such a way that among women who reported higher marital intimacy, life satisfaction was higher. Previous studies have consistently reported a positive association between marital intimacy [44] and marital adjustment [45] with women’s life satisfaction. High sexual functioning was the other predictor of women’s life satisfaction in present study. Despite an extensive search, the relationship between sexual function and life satisfaction among women of reproductive age has not been investigated previously. However, studies among older adults [7], older men [6], and aging women [46] have consistently reported that improvement of sexual activity increases the level of life satisfaction. It also appears that there is a bidirectional relationship between women’s sexual-related determinants and life satisfaction [47].

### Limitations

The cross-sectional nature of the study, the use of self-report to complete the psychometric scales, and lack of assessing spouses simultaneously were the main limitations of the present study. Also, the participants in the study were only sampled from one urban area. While the study setting (Qazvin city) is one of main cites in Iran with cultural diversity within the population, the results cannot necessarily be generalized to the Iranian population. Further studies using a longitudinal design with participation of couples from other countries and cultures are recommended.

## Conclusion

The findings in the present study demonstrated that intimacy and good sexual functioning were the strongest predictors good life satisfaction among Iranian women. These variables can be considered in counseling sessions and designing interventions to improve these specific variables may help in increasing women’s life satisfaction.

## Data Availability

Data and materials will be available upon email to the corresponding author.
